# Classification and Temporal Variability in Urinary 8-oxodG and 8-oxoGuo: Analysis by UHPLC-MS/MS

**DOI:** 10.1038/s41598-019-44240-0

**Published:** 2019-06-03

**Authors:** Ziqi Li, Yuan Yao, Yanfei Zhang, Yining Zhang, Yijun Shao, Chuanxi Tang, Weidong Qu, Ying Zhou

**Affiliations:** 1 Centers for Water and Health, Key Laboratory of Public Health Safety, Ministry of Education, Fudan University, Shanghai, 200032 China; 2 Department of Nutrition and Food Hygiene and Chemistry, School of Public Health, Key Laboratory of Public Health Safety, Ministry of Education, Fudan University, Shanghai, 200032 China; 3 Centers for Disease Control and Prevention of Changnin distribution, Shanghai, 200050 China; 4 Department of Environmental Health, School of Public Health, Key Laboratory of Public Health Safety, Ministry of Education, Fudan University, Shanghai, 200032 China; 5 Pudong New Area for Disease Control and Prevention, Fudan University Pudong Institute of Preventive Medicine, Shanghai, 200136 China

**Keywords:** Risk factors, Biomarkers

## Abstract

Oxidative stress damage has been found to be associated with exposure of children to environmental pollutants, but there are few data on the variability of urinary oxidative stress biomarkers and the accuracy of biomarker concentration classification. We performed a longitudinal study in Chinese school-aged children to investigate the variability of urinary 8-oxo-7,8-dihydro-2′-deoxyguanosine (8-oxodG) and 8-oxo-7,8-dihydroguanosine (8-oxoGuo) concentrations and the ability of a single first morning urine sample to assess accuracy and sensitivity of biomarkers concentration classification. After adjusting for both creatinine and specific gravity, we characterized the distribution and reproducibility of repeated measurement of 8-oxodG and 8-oxoGuo by using intraclass correlation coefficients (ICCs) derived from linear mixed model and performed surrogate category analyses to determine whether a single spot sample could accurately classify 8-oxodG and 8-oxoGuo levels. Results indicated that the geometric mean (GM) concentrations of 8-oxodG and 8-oxoGuo were 3.865 ng/mL and 5.725 ng/mL, respectively. High variability of 8-oxodG and 8-oxoGuo was observed in the single spot first morning urine sample (ICC = 0.25 and 0.18, respectively). Three repeated urinary specimens achieved sensitivity of 0.87 for 8-oxodG and 0.83 for 8-oxoGuo in low tertile and sensitivity of 0.78 in high tertile. But classification in medium tertile was less accurate for both 8-oxodG and 8-oxoGuo. In conclusion, high variability of urinary 8-oxodG and 8-oxoGuo levels results in repeated samplings needed for accurate classification.

## Introduction

Oxidative stress damage in children exposed to environmental pollution has aroused great concerns worldwide^1^. For measurement of oxidative stress, urinary biomarker detection is most commonly used in population epidemiological studies for noninvasive, convenient and cost-effective characteristics^2^. 8-oxo-7,8-dihydro-2′-deoxyguanosine (8-oxodG) and 8-oxo-7,8-dihydroguanosine (8-oxoGuo) are well recognized products of oxidative modification of nitrogenous bases in DNA and RNA respectively. The abnormal levels of urinary 8-oxodG among healthy people are associated with exposure to environmental mutagens and carcinogens or unhealthy lifestyle behaviors^3,4^; levels of 8-oxoGuo in urine were associated with exposure to environmental nanomaterials in workers^5,6^. Significantly elevated levels of 8-oxoGuo were found in biological samples when the oxidative damage to RNA occurred and the oxidation of guanine residues in RNA seems to happen more frequently than that in DNA because the bases of RNA are less ‘protected’^7^.

When exploring the associations between chemical levels and adverse health effects, inaccurate concentration classification of the biomarkers that often relies on a single measurement would strongly affect the reliability of conclusions. Several studies have investigated the accuracy of exposure classifications or temporal variability on environmental pollutants including bisphenol A^8^, phenol and paraben metabolites^9^, metal levels^10^, triclosan^11^ to explore whether the spot samples could sufficiently characterize exposure assessment based on concentration classification for considering the short half-life of bisphenol A^12^, phenol^13^ and triclosan^14^ and variable exposure to metals^10^. Similar to these chemicals, 8-oxodG and 8-oxoGuo had also short half-lives, for instance the half-life of 8-oxodG was approximately 11 min for mammalian DNA^13,15^. Although in epidemiological studies 8-oxodG/8-oxoGuo concentrations based on spot samples were used to be categorized into several groups^16,17^, to the best of our knowledge, no research has been conducted to explore the accuracy of 8-oxodG/8-oxoGuo classification using a spot urine sampling in cohort study.

The 8-oxodG levels in urine can be determined by liquid chromatography with electrochemical detection (HPLC-EC)^18^, liquid chromatography with tandem mass spectrometry (LC-MS/MS)^19^, gas chromatography with mass spectrometry (GC-MS)^20^ and enzyme-linked immunosorbent assay (ELISA) kits^21^. Determination of 8-oxoGuo has been conducted by liquid chromatographic assay^21^ or by ELISA kits for serum samples^22^. Compared with ELISA method, isotope-dilution LC-MS/MS has advantages in measurement of 8-oxodG with high accuracy, specificity and sensitivity in human subjects^23^. To improve analysis sensitivity and shorten analysis time, ultrahigh-performance liquid chromatography with tandem mass spectrometry (UHPLC-MS/MS) technology has been widely used for biological sample analysis^24^.

In this study, a sensitive and rapid method based on isotope-dilution UHPLC-MS/MS was optimized for simultaneous determination of 8-oxodG and 8-oxoGuo in urinary samples. Furthermore, the urinary 8-oxodG and 8-oxoGuo levels for 70 Chinese school-aged children from a longitudinal cohort study were used for evaluation of variability and misclassification of 8-oxodG and 8-oxoGuo levels for individuals by spot urine analysis.

## Results

### Optimization of LC-MS/MS conditions

The positive ESI MRM mode was applied and the transitions from precursor molecular ions to related product ions were optimized by injecting 1 μg/mL standard solutions. The optimal MRM ion transitions conditions for 8-oxodG, [^15^N_5_]8-oxodG and 8-oxoGuo are presented in Supplementary Table S1.

8-oxodG and 8-oxoGuo can be separated on a C_18_ column. Given that organic solvent and pH value of mobile phase would affect ionization efficiency in ESI^+^-MS/MS, two organic solvents (methanol and acetonitrile) and two additives (formic acid and acetic acid) were used for optimization of mobile phase. By adding organic acid in the mobile phase, 8-oxodG and 8-oxoGuo were efficiently ionized under positive ESI conditions (data not shown). As shown in Fig. S1, maximum signal intensity for 8-oxodG and 8-oxoGuo was attained by using 92.5% water containing 0.1% acetic acid (solvent A) and 7.5% methanol (solvent B) as the mobile phase.

### Method validation

The method validation data were summarized in Table 1. The coefficients of determination (R^2^) for 8-oxodG and 8-oxoGuo were greater than 0.99 with the regression formulas, *y = 0.055x − 0.04*(8-oxodG) and *y* = *0.268x − 0.107*(8-oxoGuo) where *y* is the peak area ratio (analyte/IS) for each analyte, *x* is concentration. The LOD for 8-oxodG was 0.09 μg/L and that for 8-oxoGuo was 0.04 μg/L, and LLOQs for 8-oxodG and 8-oxoGuo were 0.32 μg/L and 0.13 μg/L, respectively. Table 1 indicates that matrix effects were reduced by isotope-labeled internal standard (IS) correction in which the matrix effects ranged from 80 to 120%.

**Table 1:** Analytical performance of the developed UPLC-MS/MS method.

Analytes	Linearity		Sensitivity		Matrix effect (%) (n = 5)	Recovery (%) (n = 5)			Precision (%) (n = 5)	
	Range (μg/L)	R^2^	LOD (μg/L)	LLOQ (μg/L)		QC (1 μg/L)	QC (5 μg/L)	QC (25 μg/L)	Intra-day	Inter-day
8-oxodG	0.50–50	0.998	0.09	0.32	57.38^a^ 105.77^b^	104.54	94.24	86.87	5.19–6.45	2.59–5.23
8-oxoGuo	0.25–25	0.994	0.04	0.13	47.81^a^ 88.49^b^	108.57	103.96	102.60	4.87–6.33	3.70–8.74

^a^Matrix effects before isotope labeled IS correction;

^b^Matrix effects after isotope labeled IS correction.

The accuracy and precision of method were evaluated at three spiked concentration levels of 1, 5 and 25 μg/L, respectively. As summarized in Table 1, the recoveries of 8-oxodG ranged from 86.87% to104.54%, and intra-day RSDs of 8-oxodG were in the range from 5.19% to 6.45%, and inter-day RSDs were from 2.59% to 5.23%; the recoveries of 8-oxoGuo ranged between 102.60% and 108.57%, and intra-day RSDs of 8-oxoGuo were in the range of 4.87% to 6.33% while inter-day RSDs were from 3.70% to 8.74%.

### Distribution and variability analysis

Urine samples collected from 70 Chinese school-aged children over a period of 240 days were analyzed by the described UPLC-MS/MS method. The detection frequency of 8-oxodG and 8-oxoGuo was 98.9% and 95.5%, respectively. The GM (geometric mean) concentrations of 8-oxodG and 8-oxoGuo were 3.865 ng/mL and 5.725 ng/mL, respectively. Positive correlations were observed between unadjusted, creatinine-adjusted and specific gravity-adjusted results of 8-oxodG and 8-oxoGuo, respectively. (Supplementary Table S2). The creatinine-adjusted and specific gravity-adjusted concentrations of 8-oxodG and 8-oxoGuo are presented in Table 2.

**Table 2:** Distribution of urinary 8-oxodG and 8-oxoGuo concentrations and intraclass correlation coefficients from 70 Chinese school children (n = 466 samples).

Biomarker	DF(%)	Unadjusted (μg/L)					Creatinine-adjusted (μg/g)					Specific gravity-adjusted (μg/L)				
		GM	Mean	Median	Max	ICC	GM	Mean	Median	Max	ICC	GM	Mean	Median	Max	ICC
8-oxodG	98.9	3.865	4.547	3.993	28.33	0.250 (0.190–0.321)^a^	4.567	5.576	4.789	31.92	0.190 (0.134–0.263)	3.837	4.494	4.041	19.00	0.240 (0.181–0.312)
8-oxoGuo	95.5	5.725	8.999	7.314	365.8	0.182 (0.127–0.255)	6.764	12.04	7.664	285.8	0.209 (0.151-0.281)	5.684	9.191	6.771	188.8	0.203 (0.146–0.276)

Abbreviations: DF, detection frequency; GM, geometric mean; Max, maximum; ICC, intraclass correlation coefficient.

^a^95% confidence interval of intraclass correlation coefficient, (lower bound, upper bound).

8-oxodG exhibited high within-child variance before urine dilution correction (ICC = 0.250, p < 0.001). And 8-oxodG concentrations were more variable after creatinine correction (ICC = 0.190, p < 0.001) than after specific gravity correction (ICC = 0.240, p < 0.001). 8-oxoGuo concentrations had poor reproducibility before urine dilution correction (ICC = 0.182, p = 0.001), but displayed similar variability both after creatinine correction (ICC = 0.209, p < 0.001) and after specific gravity correction (ICC = 0.203, p < 0.001).

Table 3 presents Pearson correlations of creatinine or specific gravity adjusted concentrations of 8-oxodG and 8-oxoGuo in repeated spot samples collected 1–240 days apart. The correlations of creatinine or specific gravity adjusted 8-oxodG levels were weak but statistically significant for samples collected on the consecutive 5 days (namely 1–4 days apart). The correlations became weaker and were not significant for samples collected 90 and 240 days apart. Similar patterns of Pearson correlations for 8-oxoGuo occurred.

**Table 3:** Pearson correlations of 8-oxodG and 8-oxoGuo concentrations (Ln scale) in paired same-child urine samples collected 1–240 days apart.

Type of analyte	Type of concentration	Days elapsed between paired samples					
		1	2	3	4	90	240
		n = 70^a^	70	70	70	64	52
8-oxodG	Creatinine-adjusted	0.358*	0.358*	0.259*	0.316*	0.035	−0.089
	Specific-gravity adjusted	0.478*	0.375*	0.420*	0.289*	0.074	−0.012
8-oxoGuo	Creatinine-adjusted	0.313*	0.341*	0.219	0.540*	−0.013	0.122
	Specific-gravity adjusted	0.374*	0.350*	0.232	0.525*	−0.076	0.180

^a^The number of within-child pairings of spot samples.

*Correlation is significant at *p* < 0.05 level.

### Surrogate category analysis

The “true” tertiles of urinary 8-oxodG and 8-oxoGuo concentration were obtained by distribution of all samples (Supplementary Table S3). For 8-oxodG, as shown in Table 4 (values in bold means values were greater than or equal to 0.80), sensitivity of the middle tertile was lower compared to the low and high tertiles in unadjusted, creatine-adjusted and specific gravity-adjusted groups. But the specificity of the middle tertile in unadjusted group, the proportion of school students who were not classified into the middle tertile when they were “truly” not in that tertile^8^, was higher than that of low and high tertile and the specificity of 8-oxodG unadjusted group in middle tertile reached the highest of 0.88 after 2 times repeated sampling. As an indicator to describe the accuracy performance of classification, positive predictive values (PPVs) (mean) of 8-oxodG in unadjusted, creatine-adjusted and specific gravity-adjusted groups ranged from 0.33 to 0.74. For 8-oxoGuo, only the sensitivity of the low tertile in unadjusted group exceeded 0.80 after 3 times random selection. Specificities in high and middle tertiles were higher than those in low tertile in unadjusted group, as in one time repeated sampling, the specificities(mean) of high and middle tertiles are 0.79 and 0.87 and the specificity(mean) of low tertile is 0.61. And PPVs (mean) of 8-oxoGuo in unadjusted, creatine-adjusted and specific gravity-adjusted groups ranged from 0.33 to 0.77.

**Table 4:** Sensitivity, specificity and PPV of urinary 8-oxodG and 8-oxoGuo concentration classification (n = 466).

Biomarker	Repeat samples	Sensitivity		Specificity		Positive Predictive Value	
		Median(IQR)	Mean(95%CI)	Median(IQR)	Mean(95%CI)	Median(IQR)	Mean(95%CI)
8-oxodG	**High tertile**						
	1	0.76(0.54,0.79)	0.68(0.50,0.86)	0.72(0.62,0.78)	0.71(0.57,0.84)	0.54(0.43,0.62)	0.53(0.40,0.65)
	2	0.74(0.71,0.78)	0.74(0.70,0.79)	**0.81(0.78,0.84)**	**0.81(0.78,0.85)**	0.67(0.62,0.69)	0.66(0.61,0.70)
	3	0.78(0.74,0.83)	0.78(0.72,0.85)	**0.85(0.74,0.89)**	**0.82(0.73,0.92)**	0.71(0.61,0.78)	0.70(0.60,0.80)
	**Middle tertile**						
	1	0.17(0.09,0.36)	0.21(0.02,0.41)	**0.84(0.77,0.92)**	**0.85(0.75.0.95)**	0.35(0.15,0.50)	0.33(0.05,0.62)
	2	0.30(0.24,0.36)	0.30(0.21,0.39)	**0.87(0.87,0.90)**	**0.88(0.86,0.91)**	0.54(0.47,0.64)	0.55(0.43,0.68)
	3	0.35(0.22,0.52)	0.37(0.18,0.55)	**0.85(0.82,0.87)**	**0.85(0.80,0.89)**	0.53(0.44,0.61)	0.52(0.40,0.64)
	**Low tertile**						
	1	0.70(0.59,0.75)	0.67(0.57,0.78)	0.77(0.66,0.79)	0.73(0.65,0.82)	0.62(0.50,0.64)	0.58(0.49,0.67)
	2	0.75(0.71,0.88)	0.78(0.67,0.90)	0.73(0.68,0.76)	0.72(0.67,0.77)	0.60(0.54,0.66)	0.60(0.52,0.67)
	3	**0.87(0.81,0.88)**	**0.85(0.80,0.89)**	**0.85(0.79,0.86)**	**0.83(0.78,0.88)**	0.73(0.69,0.75)	0.72(0.67,0.780
8-oxodG-creatinine adjusted	**High tertile**						
	1	0.59(0.51,0.63)	0.57(0.49,0.65)	0.76(0.63,0.83)	0.74(0.58,0.89)	0.54(0.45,0.89)	0.52(0.41,0.64)
	2	0.73(0.54,0.76)	0.67(0.51,0.82)	**0.89(0.83,0.89)**	**0.87(0.82,0.92)**	0.69(0.65,0.77)	0.71(0.63,0.78)
	3	0.78(0.59,0.83)	0.72(0.57,0.88)	**0.87(0.84,0.91)**	**0.88(0.83,0.92)**	0.76(0.67,0.80)	0.74(0.65,0.83)
	**Middle tertile**						
	1	0.36(0.24,0.51)	0.37(0.20,0.55)	0.74(0.69,0.82)	0.75(0.65,0.86)	0.44(0.39,0.48)	0.43(0.37,0.50)
	2	0.39(0.27,0.43)	0.36(0.22,0.50)	0.72(0.71,0.82)	0.75(0.68,0.83)	0.39(0.33,0.51)	0.42(0.27,0.56)
	3	0.39(0.37,0.46)	0.41(0.35,0.47)	0.79(0.72,0.84)	0.78(0.71,0.86)	0.47(0.45,0.53)	0.49(0.43,0.54)
	**Low tertile**						
	1	0.57(0.43,0.64)	0.54(0.51,0.67)	0.77(0.70,0.81)	0.76(0,69,0.83)	0.59(0.42,0.63)	0.54(0.40,0.67)
	2	0.71(0.62,0.75)	0.69(0.60,0.78)	0.73(0.70,0.77)	0.74(0.69,0.78)	0.57(0.54,0.62)	0.58(0.52,0.63)
	3	0.67(0.63,0.79)	0.70(0.58,0.81)	0.76(0.71,0.80)	0.76(0.67,0.84)	0.59(0.54,0.66)	0.60(0.52,0.68)
8-oxodG-specific gravity adjusted	**High Tertile**						
	1	0.50(0.45,0.64)	0.54(0.41,0.66)	0.70(0.61.0.77)	0.69(0.56,0.82)	0.43(0.38,0.54)	0.45(0.32,0.59)
	2	0.61(0.49,0.70)	0.59(0.45,0.74)	**0.82(0.81,0.86)**	**0.83(0.80,0.87)**	0.67(0.55,0.70)	0.63(0.52,0.74)
	3	0.65(0.61,0.74)	0.67(0.59,0.75)	**0.85(0.82,0.88)**	**0.85(0.81,0.93)**	0.68(0.62,0.76)	0.69(0.60,0.71)
	**Middle Tertile**						
	1	0.32(0.24,0.38)	0.31(0.19,0.42)	0.71(0.67,0.80)	0.73(0.64,0.82)	0.35(0.28,0.49)	0.38(0.21,0.54)
	2	0.35(0.31,0.45)	0.37(0.25,0.50)	0.74(0.69,0.84)	0.76(0.66,0.86)	0.46(0.36,0.50)	0.43(0.34,0.53)
	3	0.52(0.43,0.59)	0.51(0.39,0.64)	0.77(0.67,0.82)	0.75(0.64,0.86)	0.48(0.42,0.61)	0.51(0.38,0.63)
	**Low Tertile**						
	1	0.65(0.38,0.77)	0.59(0.28,0.90)	0.79(0.75,0.84)	0.79(0.72,0.86)	0.67(0.40,0.70)	0.58(0.33,0.82)
	2	**0.83(0.66,0.88)**	0.78(0.64,0.92)	0.76(0.74,0.86)	0.79(0.71,0.87)	0.66(0.60,0.73)	0.67(0.58,075)
	3	0.75(0.64,0.79)	0.72(0.61,0.83)	**0.85(0.80,0.91)**	**0.86(0.78,0.93)**	0.72(0.68,0.79)	0.73(0.65,0.81)
8-oxoGuo	**High Tertile**						
	1	0.57(0.50,0.71)	0.60(0.46,0.73)	**0.80(0.75,0.82)**	0.79(0.74,0.84)	0.56(0.53.0.60)	0.56(0.51,0.62)
	2	0.65(0.61,0.75)	0.68(0.58,0.77)	0.79(0.76,0.82)	0.79(0.75,0.83)	0.61(0.58,0.65)	0.61(0.57,0.66)
	3	0.70(0.70,0.78)	0.73(0.67,0.79)	**0.85(0.79,0.85)**	**0.86(0.77,0.94)**	0.72(0.63,0.81)	0.72(0.58,0.86)
	**Middle tertile**						
	1	0.22(0.13,0.22)	0.18(0.13,0.24)	**0.86(0.83,0.91)**	**0.87(0.82,0.92)**	0.38(0.34,0.54)	0.43(0.28,0.58)
	2	0.30(0.28,0.30)	0.30(0.27,0.32)	**0.81(0.78,0.84)**	**0.81(0.77,0.85)**	0.44(0.41,0.47)	0.44(0.39,0.49)
	3	0.39(0.30,0.41)	0.37(0.28,0.45)	**0.85(0.82,0.87)**	**0.85(0.81,0.89)**	0.53(0.49,0.59)	0.54(0.46,0.62)
	**Low tertile**						
	1	0.77(0.73,0.79)	0.76(0.72,0.80)	0.60(0.56,0.66)	0.61(0.54,0.68)	0.50(0.48,0.53)	0.50(0.47,0.54)
	2	0.79(0.64,0.81)	0.74(0.62,0.85)	0.76(0.71,0.80)	0.76(0.69,0.82)	0.60(0.58,0.65)	0.61(0.57,0.65)
	3	**0.83(0.79,0.88)**	**0.83(0.77,0.90)**	0.78(0.71,0.82)	0.77(0.68,0.85)	0.66(0.59,0.71)	0.65(0.57,0.74)
8-oxoGuo creatinine adjusted	**High tertile**						
	1	0.55(0.39,0.61)	0.51(0.37,0.65)	**0.84(0.74,0.85)**	**0.80(0.72,0.89)**	0.53(0.46,0.63)	0.54(0.44,0.65)
	2	0.65(0.59.0.78)	0.68(0.54,0.81)	**0.83(0.79,0.89)**	**0.84(0.76.0.91)**	0.65(0.61,0.77)	0.68(0.58,0.78)
	3	0.78(0.72,0.87)	0.79(0.67,0.91)	**0.89(0.84,0.91)**	0.88(0.83,0.93)	0.78(0.71,0.81)	0.77(0.71,0.83)
	**Middle tertile**						
	1	0.23(0.16,0.38)	0.26(0.12,0.40)	0.77(0.70,0.78)	0.74(0.69,0.80)	0.33(0.26,0.40)	0.33(0.24,0.42)
	2	0.39(0.35,0.50)	0.42(0.32,0.52)	0.78(0.73,0.83)	0.78(0.72,0.85)	0.47(0.43,0.57)	0.49(0.40,0.59)
	3	0.48(0.37,0.57)	0.47(0.35,0.59)	**0.81(0.76,0.88)**	**0.82(0.72,0.91)**	0.50(0.48,0.69)	0.57(0.41,0.72)
	**Low tertile**						
	1	0.65(0.60,0.72)	0.66(0.56,0.76)	0.65(0.63,0.72)	0.67(0.61,0.73)	0.52(0.47,0.56)	0.52(0.46,0.57)
	2	0.71(0.63,0.81)	0.72(0.59,0.84)	**0.80(0.74,0.82)**	0.78(0.73,0.83)	0.63(0.61,0.67)	0.64(0.60,0.68)
	3	0.79(071,0.83)	0.78(0.69,0.86)	0.78(0.78,0.88)	**0.82(0.75,0.89)**	0.66(0.64,0.77)	0.70(0.59,0.80)
8-oxoGuo specific gravity adjusted	**High tertile**						
	1	0.63(0.50,0.70)	0.61(0.48,0.73)	0.75(0.68,0.80)	0.74(0.66,0.82)	0.50(0.48,0.58)	0.52(0.44,0.61)
	2	0.70(0.70,0.78)	0.73(0.67,0.78)	**0.80(0.68,0.84)**	0.77(0.66,0.88)	0.67(0.53,0.68)	0.62(0.51,0.72)
	3	0.74(0.74,0.80)	0.77(0.72,0.81)	**0.83(0.80,0.85)**	0.83(0.79,0.86)	0.68(0.65,0.71)	0.68(0.65,0.72)
	**Middle tertile**						
	1	0.13(0.11,0.18)	0.14(0.08,0.20)	**0.88(0.83,0.91)**	**0.87(0.82,0.93)**	0.33(0.28,0.50)	0.38(0.20,0.56)
	2	0.26(0.17,0.35)	0.26(0.15,0.37)	**0.85(0.85,0.93)**	**0.88(0.82,0.94)**	0.53(0.41,0.64)	0.53(0.38,0.68)
	3	0.35(0.24,0.41)	0.33(0.21,0.45)	**0.83(0.83,0.90)**	**0.86(0.81,0.91)**	0.53(0.42,0.65)	0.53(0.36,0.70)
	**Low tertile**						
	1	0.61(0.60,0.76)	0.66(0.54,0.79)	0.59(0.54,0.63)	0.59(0.52,0.65)	0.45(0.40,0.50)	0.45(0.38,0.52)
	2	0.67(0.58,0.75)	0.66(0.55,0.78)	0.65(0.63,0.74)	0.68(0.61,0.75)	0.52(0.49,0.54)	0.52(0.49,0.55)
	3	0.75(0.75,0.81)	0.78(0.73,0.82)	0.78(0.67,0.82)	0.75(0.66,0.85)	0.64(0.55,0.69)	0.63(0.53,0.72)

Abbreviations: IQR, interquartile range; CI, confidence interval.

## Discussion

In this study a sensitive isotope dilution method based on UHPLC-MS/MS was optimized for high throughput determination of two oxidative stress biomarkers, 8-oxodG and 8-oxoGuo, in urine samples collected from one longitudinal cohort study of school children during a period of nine months. The levels of 8-oxodG and 8-oxoGuo in first morning urine samples from children 7–11 years old indicated high variability. 8-oxodG and 8-oxoGuo measured in one spot urine sample have relatively low sensitivity to identify children who would be considered the most highly exposed^25^. To the best of our knowledge, it is the first time that the variability and misclassification of urinary 8-oxodG and 8-oxoGuo levels based on single spot sampling have been explored.

It is generally believed that in positive ESI-MS/MS, methanol displays great advantages over acetonitrile (ACN). This is mainly because methanol can provide protons that give rise to greater ionization efficiency in comparison to ACN. In this study we observed that the signal intensities of both 8-oxodG and 8-oxoGuo were higher in methanol mobile phase compared with ACN. Thus, methanol was chosen as organic solvent in the mobile phase. Further experiments indicated that acetic acid notably increased the signal intensity of target analytes when used as an organic modifier in mobile phase.

Matrix effects (ME) are a major concern in quantitative liquid chromatography–mass spectrometry (LC-MS) because they detrimentally affect the accuracy, reproducibility, and sensitivity of quantitative analysis^26^. ME occurs when compounds that are coeluted with the analyte interfere with the ionization process in the MS detector, thereby causing ionization suppression or enhancement. The most well-recognized technique available to correct for matrix effects is that of internal standardization using stable isotope-labeled versions of the analyte^27,28^. The O^18^ stable-labelled-8-oxodG has been used as an internal standard for the analysis of both 8-oxoGuo and 8-oxodG in urine samples^29^. In the present study, matrix effects of 8-oxodG and 8-oxoGuo corrected by [^15^N_5_] labeled 8-oxodG were compensated for up to 40% of reduction compared with those uncorrected by [^15^N_5_] labeled 8-oxodG (Table 3). In addition, sample dilution is commonly proposed as a strategy to reduce or eliminate matrix effects of urinary sample during LC-MS/MS analysis^30^. In this study a simple fivefold dilution of urine sample with ultra-pure water was used for sample preparation to further reduce the matrix effect. Furthermore, direct dilution of urine samples considerably shortened the sample pretreatment time and also avoided the loss of analytes of interest compared with solid phase extraction as reported by previous studies^31–33^.

The LOD of 8-oxodG in this study was similar to LODs measured by Cervinkova B^33^ and Ren, L^32^, but was somewhat lower than previous studies^31,34,35^. For 8-oxoGuo, the LOD of the proposed method was one order of magnitude lower than that reported by Cervinkova B^33^ and was three orders of magnitude lower than that reported by Rodríguez-Gonzalo E^35^ (Supplementary Table S4). Recoveries of 8-oxodG and 8-oxoGuo were within the range from 80 to 120% with RSDs of 2.59~8.74 indicating good accuracy and precision in accordance with European Medicine Agency (EMA) guidelines^36^. The analysis time of sample was as short as 6 minutes suggesting that this proposed method was propitious to be adopted for analysis of large numbers of urine samples.

In the present study we found intra-individual variations of 8-oxodG and 8-oxoGuo in the spot urina *sanguinis* were high during the monitoring period of nine months. The ICCs of 8-oxodG or 8-oxoGuo were less than 0.40, which implied poor reproducibility of measures of spot samples as previously reported^37^. High variations of 8-oxodG or 8-oxoGuo in spot urina sanguinis were also observed in other researches. Urinary biomarkers of oxidative stress were measured in samples from 4 study visits across pregnancy indicating variable unadjusted 8-oxodG concentrations with ICC = 0.32^38^. Saliva of healthy adults was collected over 3 consecutive days to assess unadjusted 8-oxodG diurnal variations and intra-individual CVs of 40% samples was higher than 35%, which was calculated by the following equation, (between-day SD/between-day mean) × 100, indicating high intra-individual variability^39^. SG and creatinine were proved to be temporal consistent in urina sanguinis collected from 243 subjects over 5 consecutive weekdays^40^. Thus, we suggested the intra-individual variations of 8-oxodG and 8-oxoGuo levels in repeated spot urina sanguinis originated from 8-oxodG and 8-oxoGuo concentration in spot urines other than the correction by creatinine and specific gravity. In addition, the weak Pearson correlations of 8-oxodG and 8-oxoGuo concentrations in repeated spot samples collected 4 apart, 90 apart and 240 apart suggested that the intra-individual variations of urinary 8-oxodG and 8-oxoGuo levels were high among seasons.

When samples were randomly selected to correctly predict a participant’s “true” tertile of urinary oxidative stress biomarkers classification, we found that the sensitivities of a single sample to predict the middle tertile of 8-oxodG and 8-oxoGuo were lower than 0.38 whether urinary dilution was corrected or not. It suggested that when concentrations of 8-oxodG and 8-oxodG were divided into tertiles based on a spot sample, the misclassification would occur for poor sensitivities. However, the sensitivities and PPVs of 8-oxodG and 8-oxoGuo had an increased tendency with the increasement of sampling times. For example, when 3 repeat samples were randomly selected, the sensitivities of unadjusted 8-oxodG and 8-oxoGuo in low tertile reached 0.85(mean) and 0.83(mean), respectively. Similar results were observed in a study on exposure classification of urinary bisphenol A^8^. Misclassification could be avoided by increasing sampling times. A single spot first morning urine sample may lead to mistakes when judging 8-oxodG/8-oxoGuo as a predictor of diseases^41^.

The main limitation of this study may be limited urine samples collected from 70 children. Previous studies of BPA exposure classification recruited 166 adults and detected 2632 urine samples to conduct classification analysis, so they had adequate samples to divide into two groups, one group was used to calculate the surrogate tertile, another was used to evaluate the “true” GM^8^. But in this study, the repeat samples used in the surrogate tertiles were also used in the calculation of the “true” GM. In this case the over inflation may occur in calculating the specificity, sensitivity and PPV.

In summary, for large scale population monitoring on urinary 8-oxodG or 8-oxoGuo levels, this proposed UHPLC-MS/MS method is recommended as alternative for high-through detection with short run time and high sensitivity. Spot first morning urine samples collected over days have high intra-individual variations in urinary 8-oxodG and 8-oxoGuo concentration. A single spot first morning urine sample could affect the classification results of 8-oxodG/8-oxoGuo. Repeated collecting samples to monitor concentrations of 8-oxodG and 8-oxoGuo are suggested to take for achieving accurate classification of oxidative stress levels. Also a larger population needs to be analyzed to conduct a more robust conclusion for this results may only support such sample collection procedure.

## Materials and Methods

### Study population and sample collection

The set of population were recruited from 70 primary school students (37 male and 33 female) aged 8~10 years (mean age, 8.63 ± 0.765 years) in Shanghai. They provided first morning urines using 50 mL polypropylene tubes during 5 consecutive weekdays of March, 2013 and one weekday of June and November, 2013. Total of 466 urine samples (including 350 urine samples from 5 consecutive weekdays of March, 2013, 64 urine samples from one weekday of June, 2013, and 52 urine samples from one weekday of November, 2013) were collected and transported to our laboratory on ice and then stored at −80 °C until for analysis. Each participant gave written informed consent before participation. The study protocol was supported by the Institutional Review Board (IRB) of the School of Public Health, Fudan University. Informed consents have been obtained from children’s parents or their legal guardians.

### Chemicals and reagents

8-oxodG standard was purchased from Sigma-Aldrich (St. Louis, MO, USA). 8-oxoGuo standard was obtained from Toronto Research Chemicals (Downsview, ON, Canada). [^15^N_5_]8-oxodG internal standard (IS)^42,43^ was from Cambridge Isotope Laboratories (Andover, MA, USA). Compounds information are listed in Table 5. The UHPLC/MS grade formic acid, acetic acid, methanol, acetonitrile (ACN) and water were purchased from Sigma-Aldrich (St. Louis, MO, USA) or Fisher Scientific (Fair Lawn, NJ, USA). All other chemicals were of at least analytical grade and from Sigma-Aldrich (St Louis, MO, USA).

**Table 5:** Physicochemical properties and structures of targeted analytes.

Analytes	Abbreviation	CAS number	Molecular weight	Log K_ow_^a^	pK_a_^b^
8-oxo-7,8-dihydro-2′-deoxyguanosine	8-oxodG	88847–89–6	283.0916	−3.83	10.09
8-oxo-7,8-dihydroguanosine	8-oxoGuo	3868–31–3	299.0865	−4.49	7.53

^a^Data were obtained from Human Metabolome Database, Chemspider;

^b^Data were from Human Metabolome Database.

### Urine creatinine and specific gravity (SG) measurements

Creatinine was measured by following the national standard method WS/T 98–1996 based on HPLC system with UV detector at 254 nm^44^. In brief, a 20 μL aliquot of urine was diluted with mobile phase to 4 mL. The mixture was thoroughly vortexed for approximately 1 min before analysis. An Agilent 1260 series high performance liquid chromatography system was used for LC separation with an XBridge 3.5 μm-C18 column (4.6 × 250 mm, Waters) operated at room temperature. The mobile phase consisted of 95% 0.05 mol/L sodium acetate and 5% methanol. The flow rate was 0.9 mL/min and the injection volume 10 μL. Calibration standards at concentrations of 0, 2, 4, 6, 8, 10 μg/mL were prepared by dissolving standard chemicals in mobile phase.

SG was measured by a digital handheld refractometer (Atago PAL 10-S, Tokyo, Japan). The range of measurement was from 1.000 to 1.060 with resolution of 0.001. The prism was cleaned by dripping distilled water after measuring each urine sample. Zero setting was performed for every 20 urine samples.

### Synthetic urine preparation

Synthetic urine was prepared by following the formula reported by Bruno Alves Rochaetal *et al*.^45^ and Uzqueda *et al*.^46^. In brief, we dissolved 3.8 g of potassium chloride, 8.5 g of sodium chloride, 24.5 g of urea, 1.03 g of citric acid, 0.34 g of ascorbic acid, 1.18 g of potassium phosphate, 1.4 g of creatinine, 0.64 g of sodium hydroxide, 0.47 g of sodium bicarbonate and 0.28 mL of sulfuric acid into 500 mL ultrapure water and then sonicated them 60 min. The synthetic urine was aliquoted into 50 mL plastic centrifuge tubes and stored at −20 °C until use.

### Stock solutions, calibration solutions and quality control samples

Standard **s**tock solutions of 8-oxodG and 8-oxoGuo were gravimetrically prepared in methanol at concentrations of 200 μg/mL and 500 μg/mL, respectively. Working standard solutions of 8-oxodG and 8-oxoGuo (1 μg/mL) were made by diluting standard stock solutions with methanol. Standard stock solutions of [^15^N_5_]8-oxodG (20 μg/mL) were prepared gravimetrically in methanol followed dilution with methanol to prepare working solutions (1 μg/mL). All standard stock solutions and standard working solutions were stored at −20 °C.

Calibration standards at concentrations of 0.25, 0.50, 1, 5, 10, 25, 50 ng/mL were prepared by spiking synthetic urine with appropriate volumes of individual standard working of analyte and IS. Quality control (QC) samples at three concentration levels (1, 5 and 15 ng/mL) were prepared by diluting standard working solutions with synthetic urine. The calibration solutions and QC were stored at 4 °C.

### Sample preparation

A 500 μL aliquot of urine was fortified with 20 μL of [^15^N_5_]8-oxodG solution (500 ng/mL) in a 10 mL volumetric flask followed by dilution with pure water (18.2 MΩ) to 2.5 mL. The mixture was thoroughly vortexed for approximately 1 min and then filtered through a 0.22-μm filter before being transferred to glass vials for analysis

### UPLC-MS/MS condition

An ultrahigh performance liquid chromatography (UPLC) Nexera X2 (Shimadzu, Kyoto, Japan) system was used for LC separation with an ACQUITY UPLC BEH 1.7 μm-C18 column (2.1 × 100 mm, Waters) operated at 40 °C. The flow rate of the mobile phase was 0.2 mL/min, and the injection volume was 10 μL. The mobile phases consisted of 92.5% water, 0.1% acetic acid (solvent A) and 7.5% methanol (solvent B). The API 8050 tandem mass spectrometer (Shimadzu, Kyoto, Japan) coupled to an electrospray ionization (ESI) source was used for quantification of each analyte. The positive ESI multiple reaction monitoring (MRM) mode was used at optimal conditions as follows: capillary voltage, 4.0 kV; nebulizing gas flow rate, 3 L/min; drying gas flow rate, 10 L/min; heating gas flow rate, 10 L/min; desolvation line temperature, 250 °C; heating block temperature, 400 °C.

### Method validation

The method validation was performed by using calibration standards and QC samples. The linear range and coefficient of determination (R^2^) were used to assess the linear regression model. Limit of detection (LOD) and lower limit of quantitation (LLOQ) were measured as (3.3σ)/S’ and (10σ)/S’ (σ is the standard deviation; S’ is slope), respectively. The accuracy and precision were evaluated by analyzing replicates of QC sample at low (1 μg/L), middle (5 μg/L) and high (25 μg/L) concentrations. The matrix effect was calculated according to the following equation^47^: Matrix effect (%) = (slope of working curve obtained by spiked synthetic urine)/(slope of standard curve)] × 100%.

### Real sample analysis

Sample preparation as described above. As for quality control of analysis procedure, three QC samples, a reagent blank sample and a procedural blank sample were injected into every batch of 20 samples.

### Variability analysis and surrogate category analysis

Analysis was performed by SPSS (Version22, IBM, Chicago, IL). All of urinary 8-oxodG and 8-oxoGuo concentrations were natural log-transformed to account for non-normal distribution. We used mixed random effects models to compute the intraclass correlation coefficient (ICC) to quantitatively assess between- and within-subject variance of urinary 8-oxodG and 8-oxoGuo concentrations. In the mixed random effects models, the identification number of participants was specified as subject variable and levels of urinary 8-oxodG or 8-oxoGuo as dependent variable. Restricted maximum likelihood was selected to estimate the covariance parameters. ICC is a measure of reproducibility of replicate measures from the same subject, defined as the ratio of between-subject variance to total variance, which ranges from 0 to 1. When ICC approaches to zero, the subjects show poor reproducibility and large within-subject variability. ICCs were classified according to the guideline proposed by Rosner: <0.4, poor; ≤0.4 to <0.75, fair to good; ≥0.75, excellent^37^.

Surrogate category analysis was performed to determine if a single first-morning urine sample could adequately describe a children’s “true” category of oxidative stress biomarkers. Firstly, we regarded the GM of 8-oxodG and 8-oxoGuo concentrations for each participant as the participant’s “true” level of oxidative stress. By dividing GM concentration into low, medium and high index tertiles for 8-oxodG and 8-oxoGuo, we classified participants into three groups. After that, we randomly selected samples from each participant’s replicates of urine samples, and we classified participants into surrogate tertiles of 8-oxodG/8-oxoGuo concentrations. By calculating the sensitivity, specificity and PPV based on the classification results from random selection, the reliability of classification would be evaluated.

## Supplementary information

Classification and Temporal Variability in Urinary 8-oxodG and 8-oxoGuo: Analysis by UHPLC-MS/MS
