# Supplementary material for: Classification and Temporal Variability in Urinary 8-oxodG and 8-oxoGuo: Analysis by UHPLC-MS/MS
Source: Sci Rep. 2019 Jun 3;9:8187. doi: 10.1038/s41598-019-44240-0 (PMC6547699; doi:10.1038/s41598-019-44240-0)
Supplement: Supplementary file 1 — Classification and Temporal Variability in Urinary 8-oxodG and 8-oxoGuo: Analysis by UHPLC-MS/MS [file 41598_2019_44240_MOESM1_ESM.docx]

**Classification and Temporal Variability in Urinary 8-oxodG and 8-oxoGuo: Analysis by UHPLC-MS/MS**

**Ziqi Li^ab^, Yuan Yao^ab^, Yanfei Zhang^ab^, Yining Zhang^ab^, Yijun Shao^ab^,** **Chuanxi Tang ^c^,** **Weidong Qu^ad^, Ying Zhou^abe^***

*a Centers for Water and Health, Key Laboratory of Public Health Safety, Ministry of Education, Fudan University, Shanghai 200032, China*

*b Department of Nutrition and Food Hygiene and Chemistry, School of Public Health, Key Laboratory of Public Health Safety, Ministry of Education, Fudan University, Shanghai 200032, China*

*c Centers for Disease Control and Prevention of Changnin distribution, Shanghai 200050, China*

*d Department of Environmental Health, School of Public Health, Key Laboratory of Public Health Safety, Ministry of Education, Fudan University, Shanghai 200032, China*

*e Pudong New Area for Disease Control and Prevention, Fudan University Pudong Institute of Preventive Medicine, Shanghai 200136, China*

* Correspondence to: Y. Zhou, Department of Nutrition and Food Hygiene and Chemistry, School of Public Health, Key Laboratory of Public Health Safety, Ministry of Education, Fudan University, Shanghai 200032, China

E-mail addresses: [yingchou@fudan.edu.cn](mailto:yingchou@fudan.edu.cn) (Y. Zhou)

**Supplementary**

**Fig. S1. Effect of mobile phase components on the signal intensity of 8-oxodG and 8-oxoGuo in the ratio of 92.5:7.5 (A/B, v/v).** A) 0.1% formic acid (A) and methanol (B); B) 0.1% acetic acid (A) and methanol (B); C) 0.1% formic acid(A) and acetonitrile (B); D) 0.1%acetic acid (A) and acetonitrile (B)

**Table S1. Chromatographic and Optimized MRM Transition Parameters of Analytes**

| **Analytes** | **Retention time (min)** | **Precursor ion (m/z)** | **Product ion (m/z)** | **Q1 Pre Bias (V)** | **CE (V)** | **Q3 Pre Bias (V)** |
| --- | --- | --- | --- | --- | --- | --- |
| 8-oxodG | 3.803 | 284.10 | 168.05^a^ | -10.0 | -13.0 | -17.0 |
|  |  |  | 140.05^b^ | -10.0 | -30.0 | -14.0 |
| [^15^N_5_]8- | 3.793 | 289.05 | 173.05^a^ | -10.0 | -12.0 | -18.0 |
| oxodG |  |  | 145.00^b^ | -10.0 | -29.0 | -29.0 |
| 8-oxoGuo | 2.659 | 300.05 | 168.05^a^ | -14.0 | -14.0 | -17.0 |
|  |  |  | 140.00^b^ | -14.0 | -30.0 | -14.0 |

**a. quantitative ion;**

**b. qualitative ion.**

**Table S2. Spearman’s correlation between unadjusted and adjusted results**

| **Analytes** | **Day** | **unadjusted and creatinine-adjusted** | **unadjusted and specific gravity-adjusted** | **creatinine-adjusted and specific gravity-adjusted** |
| --- | --- | --- | --- | --- |
| **8-oxodG** |  |  |  |  |
|  | 1 | 0.640* | 0.765* | 0.851* |
|  | 2 | 0.308* | 0.615* | 0.759* |
|  | 3 | 0.378* | 0.590* | 0.831* |
|  | 4 | 0.455* | 0.677* | 0.862* |
|  | 5 | 0.423* | 0.682* | 0.722* |
|  | 6 | 0.186 | 0.517* | 0.691* |
|  | 7 | 0.590* | 0.691* | 0.595* |
| **8-oxoGuo** |  |  |  |  |
|  | 1 | 0.848* | 0.926* | 0.909* |
|  | 2 | 0.794* | 0.889* | 0.942* |
|  | 3 | 0.761* | 0.846* | 0.930* |
|  | 4 | 0.839* | 0.916* | 0.954* |
|  | 5 | 0.802* | 0.892* | 0.918* |
|  | 6 | 0.818* | 0.923* | 0.890* |
|  | 7 | 0.724* | 0.840* | 0.756* |

***. Correlation is significant at the 0.01 level (2-tailed).**

**Table S3. The range of concentrations for classification of urinary 8-oxodG and 8-oxoGuo concentrations (n=466)**

|  | **High** | **Middle** | **Low** |
| --- | --- | --- | --- |
| 8-oxodG/μg·L^-1^ | ≥4.60 | ≥3.80 to <4.60 | < 3.80 |
| 8-oxodG/Cre/μg·g^-1^ | ≥5.98 | ≥4.40 to <5.98 | < 4.40 |
| 8-oxodG/SG/μg·L^-1^ | ≥4.90 | ≥3.58 to <4.90 | < 3.58 |
| 8-oxoGuo/μg·L^-1^ | ≥9.10 | ≥6.83 to <9.10 | < 6.83 |
| 8-oxoGuo/Cre/μg·g^-1^ | ≥11.70 | ≥7.15 to <11.70 | < 7.15 |
| 8-oxoGuo/SG/μg·L^-1^ | ≥8.63 | ≥6.66 to <8.63 | < 6.66 |

**Table S4. Comparison of validation parameters from other published research in five years preceding this study**

| **Analytes**^a^ | **Volume(μL)** | **Pretreatment** | **Detection method** | **Recovery (%)** | **Precision (%)** | **LOD(ng/mL)** | **Reference** |
| --- | --- | --- | --- | --- | --- | --- | --- |
| 8-oxodG, creatinine | 100 | On-line SPE | HPLC-MS/MS | 91-101.4 | 1.7-3.1^b^  5.0-9.6^c^ | 0.32 | ^33^ |
| 8-oxodG | 10000 | MIP SPME | CE | 91 | 1.12^b^  4.03^c^ | 0.73 | ^30^ |
| 8-oxodG, 8-oxoGuo, 1mA,7mG  A, INN,  5mU, 2dG | NA | SPE  (HLB+ABN+ENV) | HPLC-MS/MS | 108-119 | 2.0-6.3^b^  5.0-10.7^c^ | 8-oxodG: 2  8-oxoGuo: 19 | ^34^ |
| 8-oxodG | 250 | On-line SPE | HPLC-MS/MS | 110.2-119.4 | 2.7-3.0^b^  4.0-4.5^c^ | 0.008 | ^45^ |
| 8-oxodG, BPA, TCS | 4000 | SPE(C_18_) | HPLC-MS/MS | 89-105.1 | 3.1-9.0^b^  4.5-8.5^c^ | 0.05 | ^31^ |
| 8-oxodG, 8-oxoGuo,  creatinine | 500 | SPE(HLB) | UHPLC-MS/MS | 98.14-109.26 | 3.20-8.83^b^  3.44-8.15^c^ | 8-oxodG: 0.11  8-oxoGuo: 0.49 | ^32^ |
| 8-oxodG, 8-oxoGuo | 500 | Direct dilution | UHPLC-MS/MS | 86.87-108.57 | 4.87-6.45^b^  2.59-8.74^c^ | 8-oxodG: 0.09  8-oxoGuo: 0.04 | This study |

**a. All analytes were listed while recovery, precision, LODs only referred to 8-oxodG/8-oxoGuo**

**b. Intra-day RSDs**

**c. Inter-day RSDs**

**C**

**B**

**A**

**Fig. S2 Chromatograms for standards.**

A) 8-oxodG; B) [^15^N_5_]8-oxodG; C) 8-oxoGuo

**Fig S3. Example chromatograms for a urine sample.**

A) 8-oxodG; B) [^15^N_5_]8-oxodG; C) 8-oxoGuo
